# Supplementary material for: Local cation-tuned reversible single-molecule switch in electric double layer
Source: Nat Commun. 2023 Jun 9;14:3397. doi: 10.1038/s41467-023-39206-w (PMC10256693; doi:10.1038/s41467-023-39206-w)
Supplement: Supplementary file 1 — Supplementary Information [file 41467_2023_39206_MOESM1_ESM.pdf]

Supplementary information

Local cation-tuned reversible single-molecule switch in electric double layer

Ling Tong[1,3], Zhou Yu[1,3], Yi-Jing Gao[1,2,3], Xiao-Chong Li[1], Ju-Fang Zheng[1], Yong Shao[1], Ya-Hao Wang[1,*], Xiao-Shun Zhou[1,*]

[1]Key Laboratory of the Ministry of Education for Advanced Catalysis Materials, Institute of Physical Chemistry, Zhejiang Normal University, Jinhua 321004, China

[2]Zhejiang Engineering Laboratory for Green Syntheses and Applications of Fluorine-Containing Specialty Chemicals, Institute of Advanced Fluorine-Containing Materials, Zhejiang Normal University, Jinhua 321004, China

[3] These authors contributed equally.

*E-mail: xszhou@ zjnu.edu.cn; yahaowang@zjnu.edu.cn

Contents

1 Reagents ... 3

2 Supplementary methods ... 3

Preparation of Au@SiO_2 nanoparticles ... 3

Theoretical calculations ... 3

3 Supplementary Table and Figures ... 5

Supplementary Table 1 ... 5

Supplementary Fig. 1 | The optimized structures of molecular adsorption. ... 6

Supplementary Fig. 2 | AIMD simulations ... 7

Supplementary Fig. 3 | PZC measurements ... 8

Supplementary Fig. 4 | PZC measurements ... 10

Supplementary Fig. 5 | Displacement distance distribution ... 11

Supplementary Fig. 6 | Conductance measurements in NH_4Cl solution ... 12

Supplementary Fig. 7 | Conductance measurements at negative bias voltage ... 13

Supplementary Fig. 8 | Conductance measurements without carboxyl groups ... 14

Supplementary Fig. 9 | CV measurements ... 15

Supplementary Fig. 10 | Electrochemical gating in the presence of different cations ... 16

Supplementary Fig. 11 | Concentration effect of metal cations ... 17

Supplementary Fig. 12 | Electrochemical gating without carboxyl groups ... 18

Supplementary Fig. 13 | In situ Raman measurements ... 19

Supplementary Fig. 14 | In situ Raman measurements ... 20

Supplementary Fig. 15 | Calculated Raman spectra ... 21

Supplementary Fig. 16 | Single-molecule switching performance ... 22

Supplementary Fig. 17 | Conductance measurements with lower single-range current amplifier ... 23

Supplementary Fig. 18 | Switch cycle test ... 24

Supplementary Fig. 19 | I-t test with feedback loop ... 25

Supplementary Fig. 20 | I-V test.. ... 26

References ... 27

1 Reagents

4-(Methylthio)benzoic acid, terephthalic acid and 3-(Methylsulfanyl)propanoic acid were purchased from Aladdin (Shanghai, China). The Au wire (φ0.25 mm, 99.9985%), $NaClO_4$ (99%), $HClO_4$ (50%), NaOH (95%), NaF (99%), NH_4Cl (99%), $Ca(ClO_4)_2$ (99%), $KClO_4$ (99%) and $Mg(ClO_4)_2$ (99%) were purchased from Alfa-Asia (Ward Hill, MA, USA) and used as received, and all aqueous solutions were prepared with ultrapure water (>18.2 MΩ cm).

2 Supplementary methods

Preparation of Au@SiO_2 nanoparticles

The 120 nm Au nanoparticles were prepared following a seed-mediated growth method[1]. Firstly, 0.6 mL of 1wt% sodium citrate solution was added into 20 mL of 0.01 wt% boiling $HAuCl_4$ solution with stirring to obtain 16 nm Au seeds. Then, to obtain 50 nm Au NPs, 3 mL of the as-prepared 16 nm Au NPs solution and 600 μL of 1 wt% ascorbic acid solution were added into 20 mL of 0.01 wt% sodium citrate solution in the ice-water bath. After stirring for 5 min, 1.1 mL of 1 wt% $HAuCl_4$ solution was dropwise added into the mixture and then moved to 70 °C bath for 30 min. Finally, 2 mL of the as-prepared 50nm Au seeds, 400 μL of 1wt% ascorbic acid, 100 μL of 1wt% sodium citrate solution and 20 mL of Milli-Q water were added to a flask with stirring in the ice-water bath. After 5 min, 654 μL of 1 wt% $HAuCl_4$ was dropwise added into the mixture and moved to a 70 °C bath for 30 min, and 120 nm Au NPs were obtained.

The 120 nm Au@SiO_2 nanoparticles were produced according to the following procedure[2]: 6 mL of the as-prepared 120 nm Au core solution was added into a flask with stirring for 10 min. Then, 0.2 mL of 1mM APTMS solution was added and stirred for 15 min. Next, 3.2 mL of 0.54 wt% sodium silicate solution was added into the mixture and stirred over night at room temperature. A thin pinhole-free silica shell was coated to the Au core. Finally, the solution was centrifuged at 3000 rpm/min for 10 min and washed with Milli-Q water twice for Raman experiments.

Theoretical calculations

All the theoretical calculations were performed by Vienna Ab Initio Simulation Package (VASP) software. The effects between electron exchange and correlation were described by the Perdew-Burke-Ernzerhof (PBE) functional, which was one of the most well-established the generalized gradient approximation (GGA). The interactions between electrons and ions were represented by projected augmented wave (PAW) potentials with a cutoff energy of 450 eV. The Brillouin-zone integration was approximated by 1×2×1 k-point sampling grid. The convergence criterion for the energy calculation was

set to 1.0×10^{-4} eV, while the force tolerance of structure optimization was set to 0.01 eV $Å^{-1}$. The van der Waals (vdW) interaction was described by DFT-D3 method.

The adsorption energy (E_{ad}) of 4-MTBA in different configurations by the following expression:

$$E_{ad} = E_{total} - E_{4\text{-MTBA}} - E_{complex}$$

Where E_{total} is the total energy of adsorbed 4-MTBA system, and $E_{4\text{-MTBA}}$ is the energy of the 4-MTBA molecule in the gas phase, $E_{complex}$ is the energy of Au layers and aqueous phase under solution condition.

3 Supplementary Table and Figures

Supplementary Table 1. Single-molecule conductance of 4-MTBA in different electrolyte solution and the pH of solution.

Electrolyte solution	pH	G at 0 V $\log(G/G_0)$	G at -0.5 V $\log(G/G_0)$
$NaClO_4$	5.01±0.01	-3.00±0.15	-
$KClO_4$	5.06±0.03	-3.02±0.10	-
$Ca(ClO_4)_2$	5.01±0.02	-2.75±0.22	-
$Mg(ClO_4)_2$	4.90±0.02	-2.92±0.11	-
NaF	6.56±0.01	-2.98±0.13	-
NH_4Cl	4.99±0.02	-2.73±0.14	-3.03±0.23
$HClO_4$	1.77±0.02	-2.98±0.17	-3.06±0.21

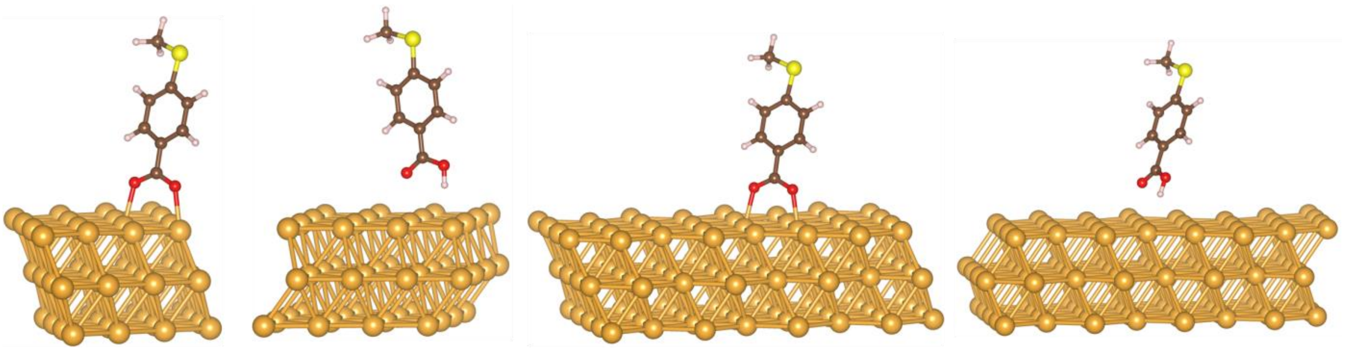

Supplementary Fig. 1 | The optimized structures of molecular adsorption. The optimized structures of adsorbed 4-MTBA with different forms of carboxyl groups on the different size of Au(111).

To verify the impact of the model, two series of Au models were constructed. As shown in Supplementary Fig. 1, a lattice cell of three-layer 2×2 Au(111) surface and three-layer 4×2 Au(111) surface with more than 15 Å vacuum layer were applied to prove the tiny influence of model size on the trend of adsorption energy. The DFT calculation results demonstrate that the adsorption of 4-MTBA with COOH (-0.60 eV) is significantly weakened than ones of 4-MTBA with COO^- (-2.11 eV) on the three-layer (2×2) Au(111) surface (48 Au atoms concluded). Certainly, the adsorption energy also exhibits the same trend on (4×2) Au(111) surface (96 Au atoms concluded), which provide solid evidence for the tiny effect of supercell size on adsorption energy.

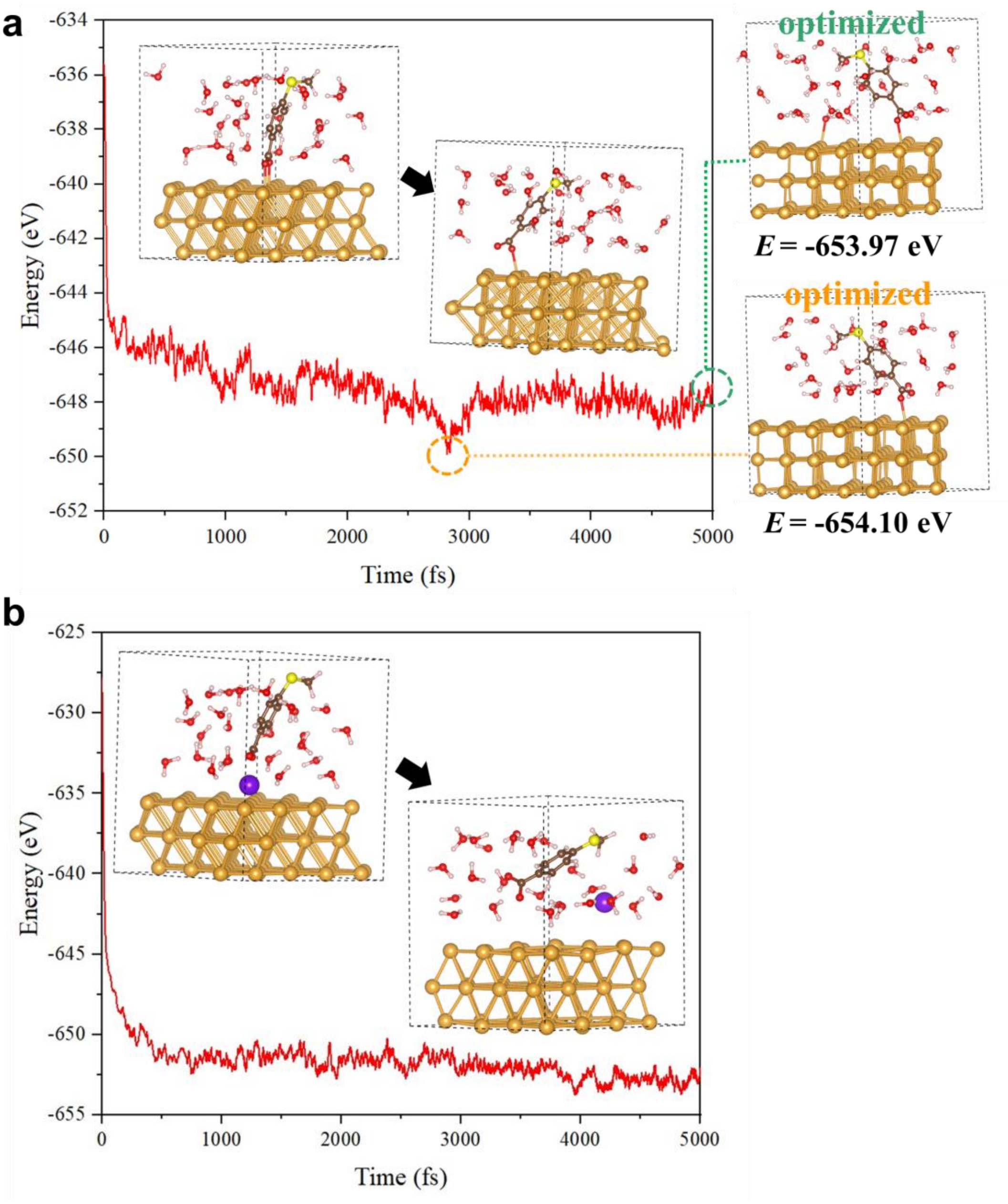

Supplementary Fig. 2 | AIMD simulations. Energy fluctuations versus AIMD simulation time (a) 4-MTBA/25H_2O/Au(111) and (b) 4-MTBA/Na-25H_2O/Au(111) at 300 K.

Supplementary Fig. 2a show the total energy against time for 4-MTBA/25H_2O/Au(111), and two optimized snapshots at 5.0 ps (green line) and 2.818 ps (orange line, lowest energy point), respectively. The geometric structures and energy of two snapshot are quite similar, indicating the structure of adsorbed 4-MTBA on the 25H_2O/Au(111) preserved well from 3 to 5 ps. Similarly, Supplementary Fig. 2b show the total energy against time for 4-MTBA/Na-25H_2O/Au(111). The energy fluctuation is almost negligible from 1 to 5 ps, indicating the structure have high thermodynamic stability. Therefore, the optimized snapshots of AIMD at 5.0 ps was applied in adsorption energy calculation.

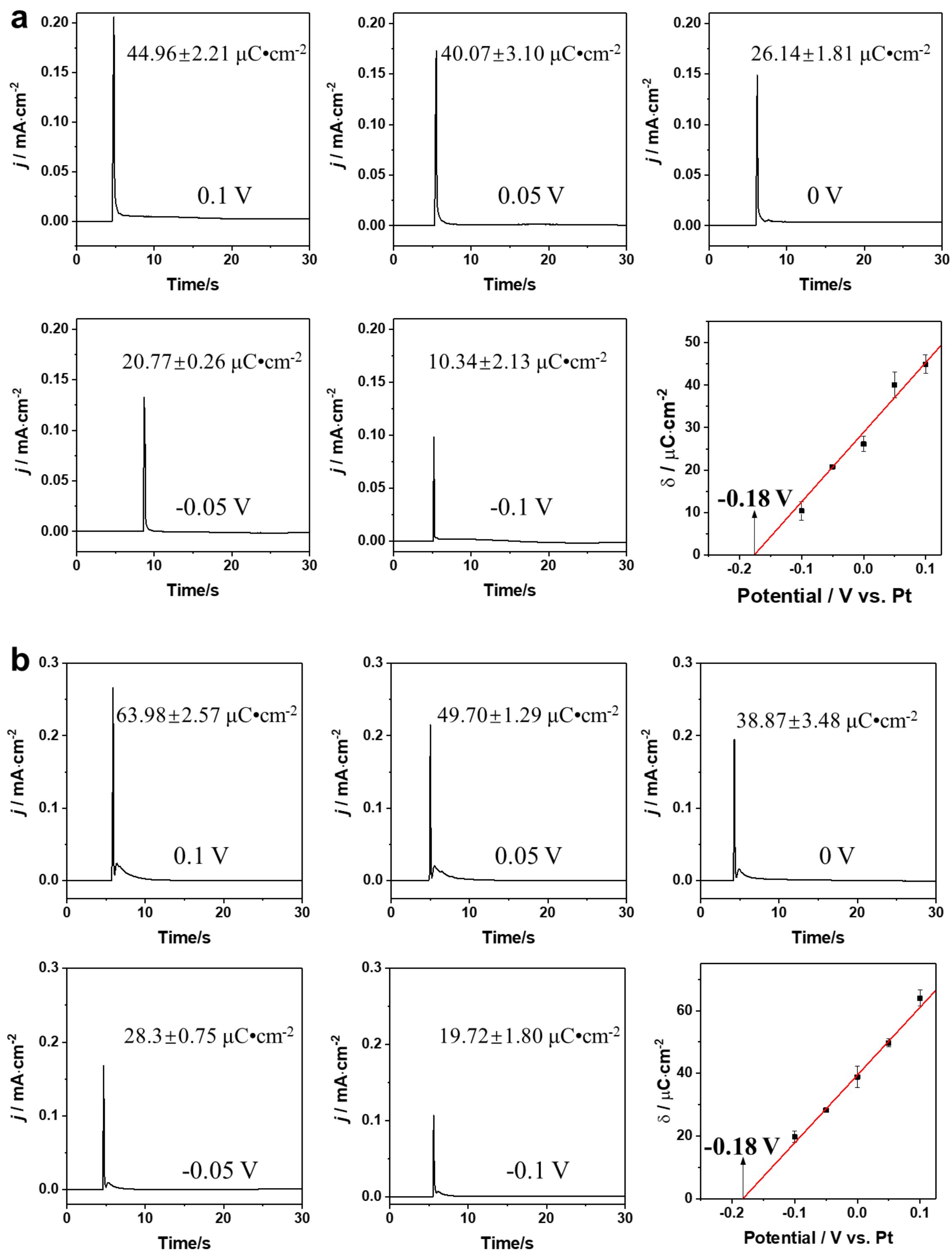

Supplementary Fig. 3 | PZC measurements. Current transients measured during the potentiostatic immersion of a Au(111) electrode in (a) 50 mM $NaClO_4$ and (b) 0.1 mM 4-MTBA + 50 mM $NaClO_4$ with control of different potentials.

According to previous report[3], ex situ immersion i-t curves were used to measure the PZC. We firstly measured the PZC of bare Au(111) in 50 mM $NaClO_4$ solution, the current transients during potential-controlled immersion experiments were shown in Supplementary Fig. 2a, the PZC are abscissa intercept

deduced from the plot of the charge density vs. potential curve, ca. -0.18 V vs. Pt for bare Au(111) in 50 mM $NaClO_4$. This is corresponding to 0.3 V vs. SCE, very close to the reported value of 0.285 V vs. SCE for Au(111) electrode

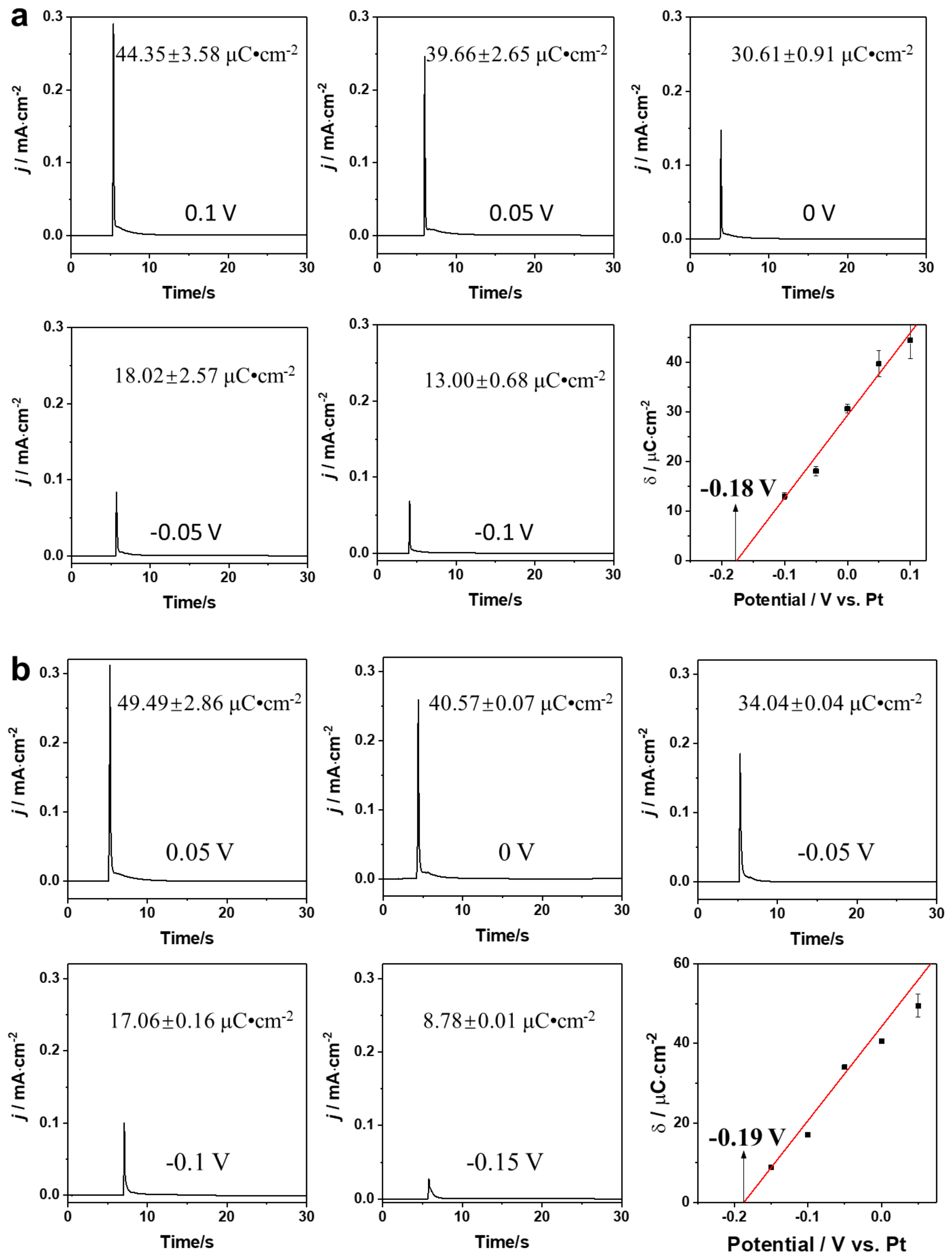

Supplementary Fig. 4 | PZC measurements. The charge density derived from integration of the immersion current transients against the applied potentials of Au(111) in 0.1 mM 4-MTBA + (a) 50 mM $HClO_4$ and (b) $Ca(ClO_4)_2$.

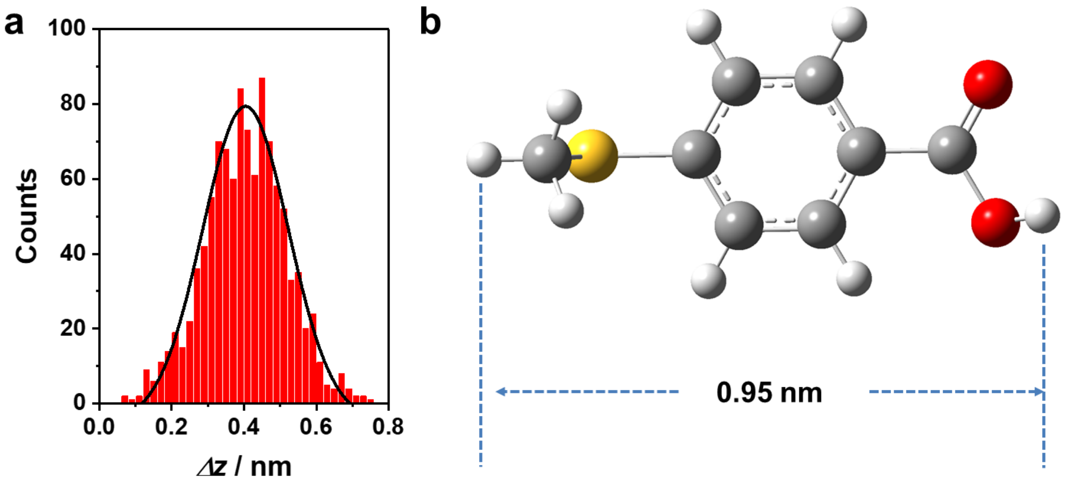

Supplementary Fig. 5 | Displacement distance distribution. Gaussian fitting of displacement distance (Δz) distribution

As shown in Supplementary Fig. 5, the step stretching distance (Δz) distribution can be Gaussian fitted into a main peak at 0.42 nm. By adding the snapback distance of breaking Au−Au contacts to the relative displacement Δz, it can be found that the most probable absolute displacement for the stretching process is 0.92 nm, which is comparable to the length of isolated 4-MTBA optimized by DFT/ B3LYP method with 6-311+G (d, p) basis sets via the Gaussian 09 software package. This confirms the formation of single-molecule junctions.

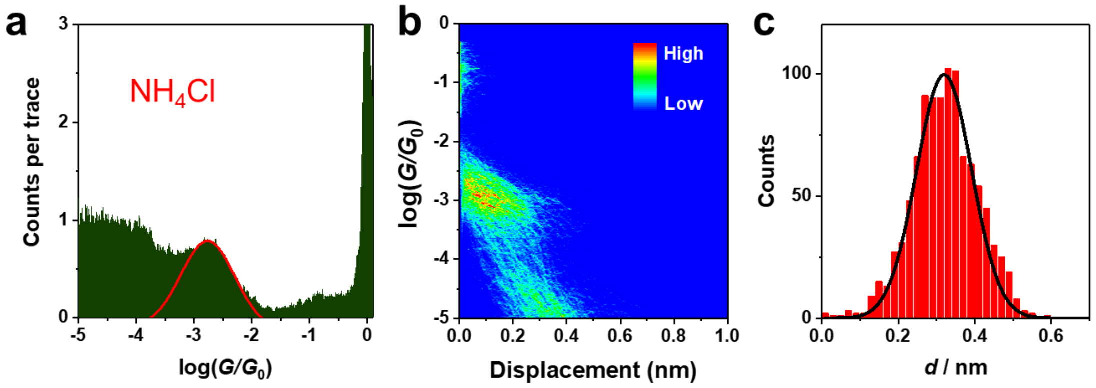

Supplementary Fig. 6 | Conductance measurements in NH_4Cl solution. (a) 1D and (b) 2D conductance histograms of 4-MTBA in NH_4Cl solution. (c) Gaussian fitting of displacement distance (Δz) distribution.

Supplementary Fig. 6a and b show 1D and 2D conductance histograms, it can be found a conductance peak and an obvious stretching states centered around $10^{-3.0}$ G_0. There may be two main reasons for the weaker conductance peak intensity in NH_4Cl solution compared to other electrolyte solutions: (1) Hydrolysis reaction $NH_4^+ + RCOO^- + H_2O \leftrightharpoons NH_3 \cdot H_2O + RCOOH$ promotes the protonation of the carboxylic acid, which decreases the junction formation probability through the deprotonated $-COO^-$ groups. (2) The strong specific adsorption of chloride ions on gold surface at potential E > PZC, have been proven a significant decrease in stability of gold atomic contacts[5,6], thereby affecting the stability of molecular junctions. This can be confirmed by the shorter step stretching displacement (*Δz)* of 0.33 nm in Fig. R11c compared to that 0.42 in $NaClO_4$ solution.

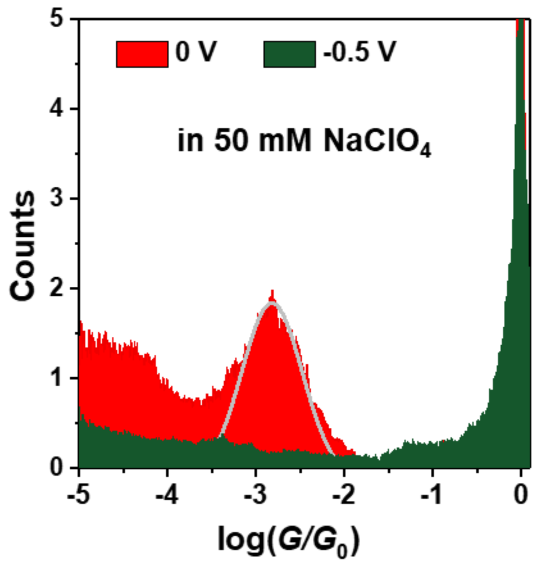

Supplementary Fig. 7 | Conductance measurements at negative bias voltage. 1D conductance histograms of 4-MTBA obtained in 50 mM $NaClO_4$ solution at 0 V and -0.5 V with a bias voltage of -50 mV. The counts are normalized by the numbers of conductance curves used. All potentials are specified with respect to Pt.

As shown in Supplementary Fig. 7, an obvious conductance peak at $10^{-2.9}$ G_0 can be observed at 0 V, while it disappears at -0.5 V, similar to that with a positive bias voltage of 50 mV in Fig. 2c. Therefore, the potential-controlled conductance switch can still work when changing the polarity of the bias voltage.

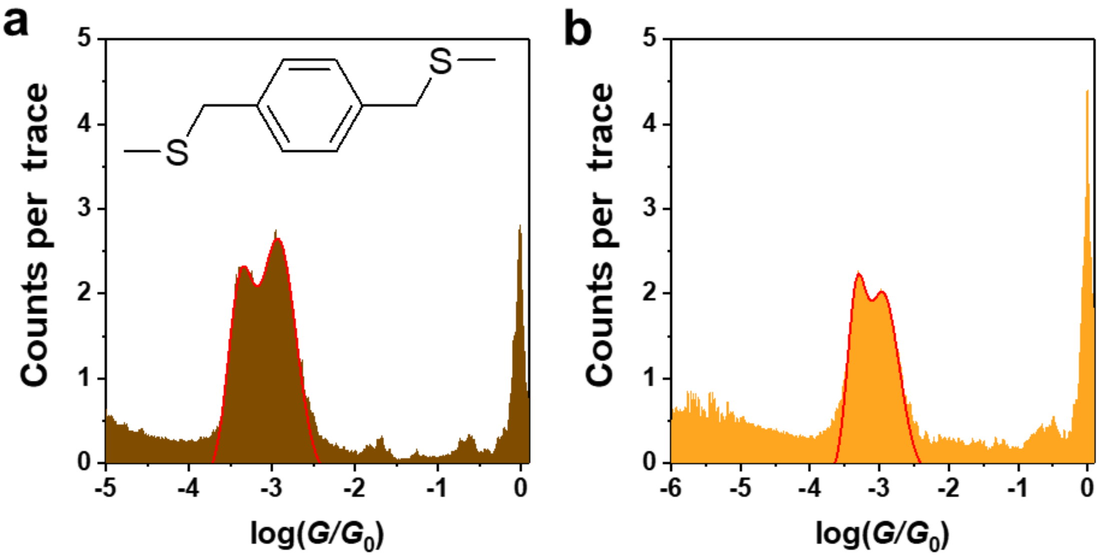

Supplementary Fig. 8 | Conductance measurements without carboxyl groups. 1D conductance histograms of 1,4-bis(methylsulfanylmethyl)benzene molecular junctions with potential control of Au(111) substrates at (a) 0 V and (b) -0.5 V vs. Pt. Insert is the molecular structure of 1,4-bis(methylsulfanylmethyl)benzene.

We also carried out the single-molecule conductance measuremnts of 1,4-bis(methylsulfanylmethyl)benzene that has two sulfide (SMe) groups at 0 and -0.5 V vs. Pt. As shown in Supplementary Fig. 8, there are two obvious conductance peaks at $10^{-3.3}$ and $10^{-2.9}$ G_0, respectively This indicates that there are two main configurations during the formation of single-molecule junctions, consistent with previous reports[4]. It can be found that changing the charge state of Au(111) with the applied potentials has a little impact on the two conductance peaks.

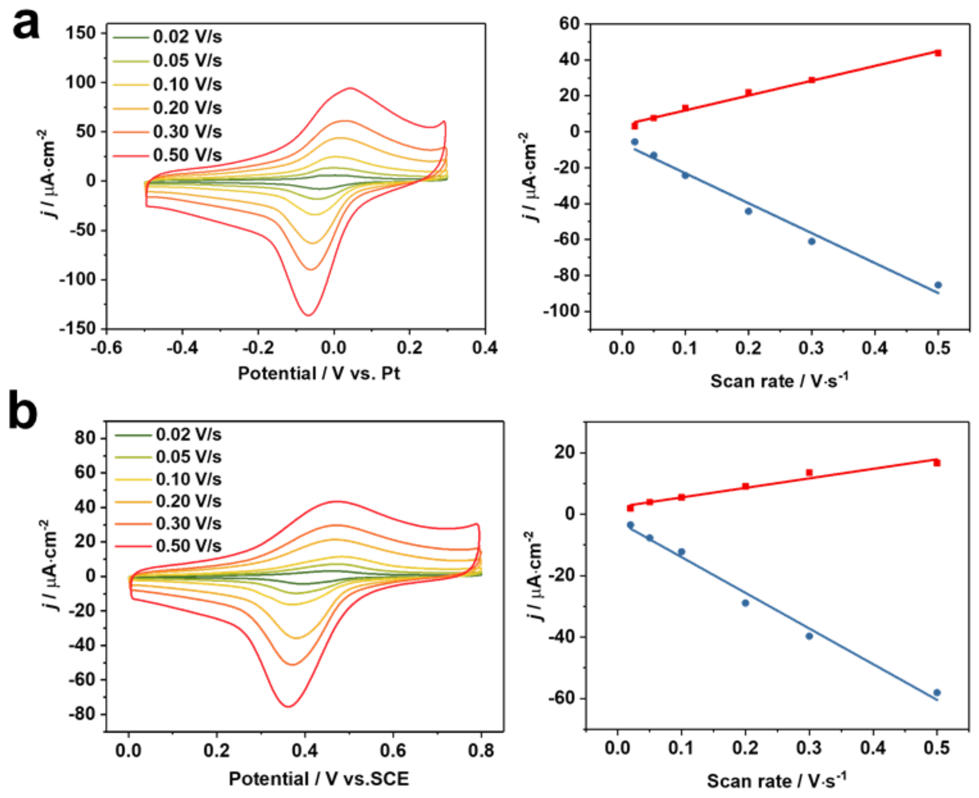

Supplementary Fig. 9 | CV measurements. CVs of Au(111) obtained in (a) 0.1 mM 4-MTBA + 50 mM $HClO_4$ solution vs. Pt and (b) 0.1 mM 4-MTBA + 50 mM $NaClO_4$ solution vs. SCE. The plot of the current density of the anodic (red square) and cathodic (blue circle) peaks against the scan rates (right panel).

We have performed CVs of Au (111) in 50 mM $HClO_4$ solution. As shown in Supplementary Fig. 9a, there is one pair of well-defined reversible peaks at about 0.05 V. A linear correlation between current density of oxidation (red square) or reduction (blue square) with the scan rates is found. This proves that the reversible peaks arise from the 4-MTBA assembled on the Au(111) interface, which is similar to the 4-MTBA assembled on the Au(111) in 50 mM $NaClO_4$ solution. We have also performed the CVs of Au(111) in 0.1 mM 4-MTBA +50 mM $NaClO_4$ solution with different scan rates using SCE as the reference electrode. As shown in Supplementary Fig. 9b, there is one pair of well-defined reversible peaks at about 0.4 V vs. SCE. Plotting current density of oxidation (red square) or reduction (blue circle) with the scan rate, a linear correlation is also observed. The total charges of oxidation peaks are quantitatively estimated at about 14.5 $\mu C/cm^2$ consistent with the results using Pt as quasi-reference electrode in Fig. 3a.

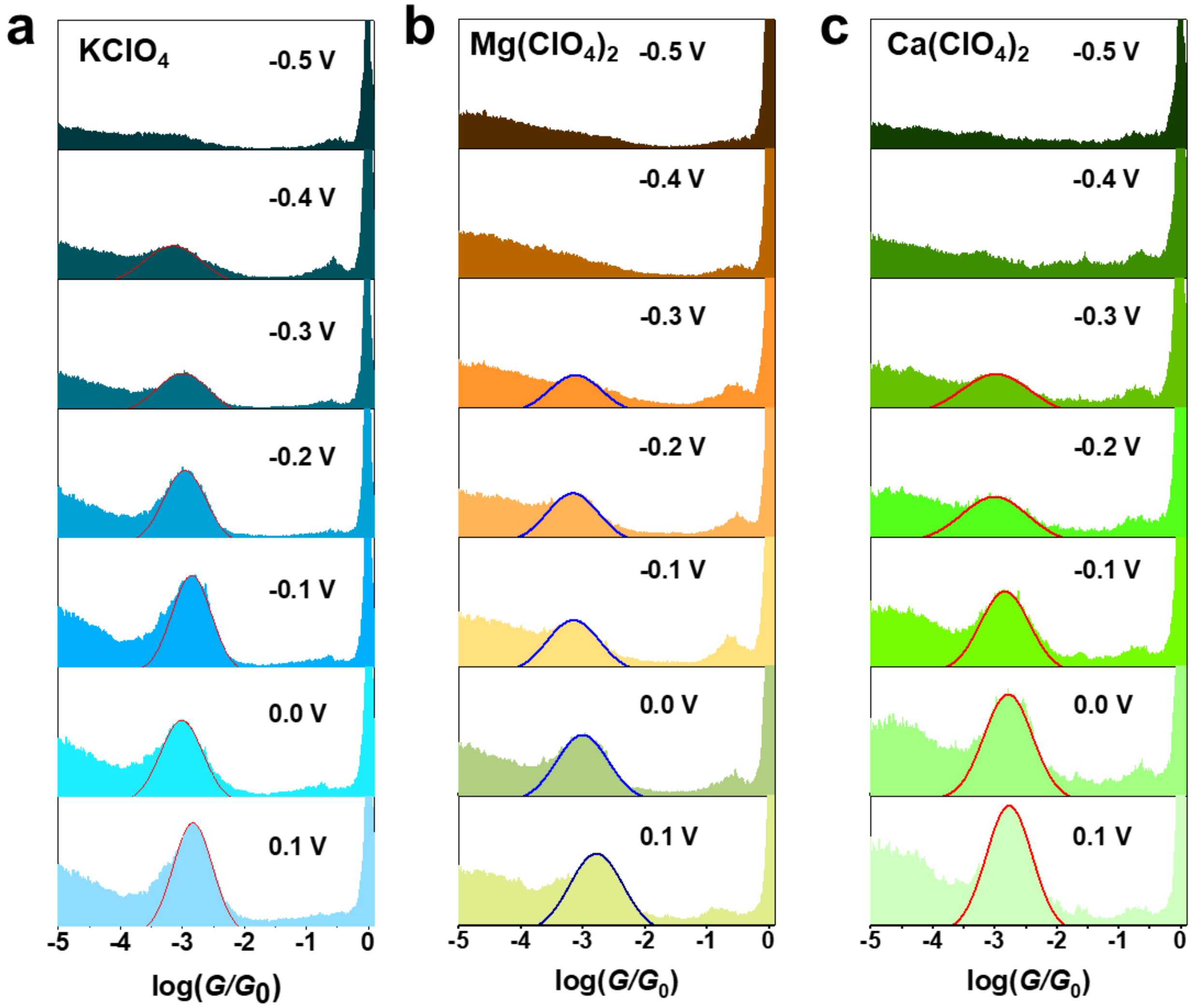

Supplementary Fig. 10 | Electrochemical gating in the presence of different cations. The potential dependent 1D conductance histogram of 4-MTBA in (a) 50 mM $KClO_4$, (b) 50 mM $Mg(ClO_4)_2$, and (c) 50 mM $Ca(ClO_4)_2$.

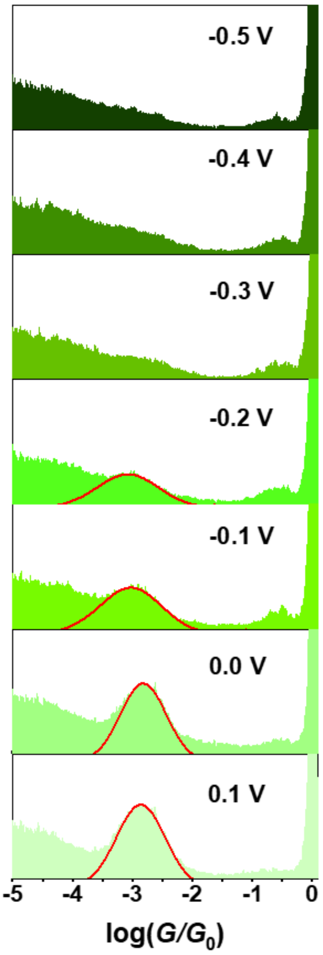

Supplementary Fig. 11 | Concentration effect of metal cations. The potential dependent 1D conductance histogram of 4-MTBA in 1 M $Ca(ClO_4)_2$. The counts are normalized by the numbers of conductance curves used. All potentials are specified with respect to Pt.

We have carried outed the electrochemical gating of single-molecule conductance measurements in 0.1 mM 4-MTBA + 1 M $Ca(ClO_4)_2$ solution. The potential-dependent 1D conductance histogram of 4-MTBA in different cations are shown in Supplementary Fig. 11. As the potential decreases, the conductance peak at $10^{-3.0}$ G_0 becomes weaker and disappears below -0.2 V in 1 M $Ca(ClO_4)_2$, which is 0.1 V earlier than in 0.05 M $Ca(ClO_4)_2$. This suggests that higher concentration of cations in the bulk solution leads to more concentrated metal cations in OHP affecting the Au-COO^- contact.

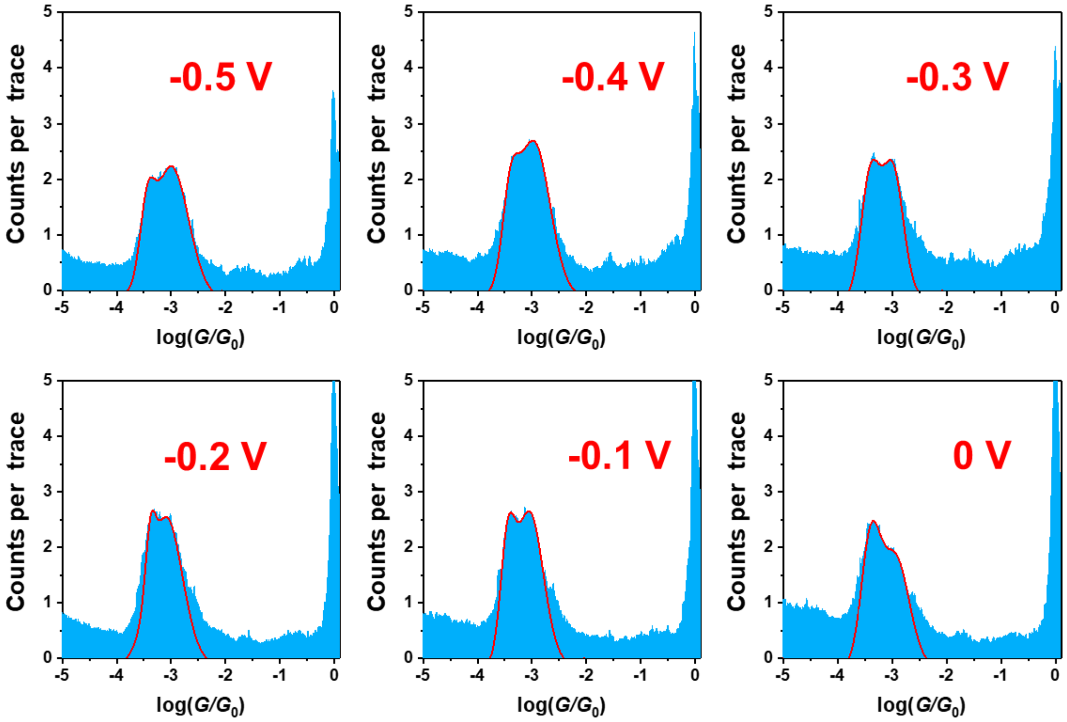

Supplementary Fig. 12 | Electrochemical gating without carboxyl groups. The potential dependent 1D conductance histogram of 1,4-bis(methylsulfanylmethyl)benzene in 50 mM $NaClO_4$.

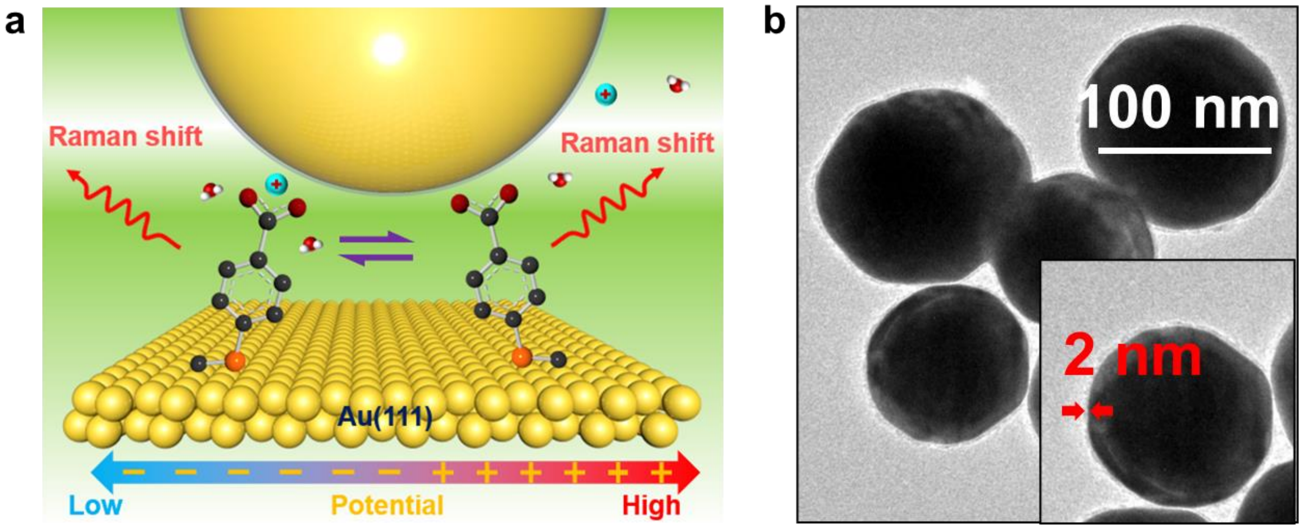

Supplementary Fig. 13 | In situ Raman measurements. (a) Schematic diagram of SHINERS technique for probing the electrochemical behavior of 0.1 mM 4-MTBA at Au(111) substrate. (b) The high-resolution TEM image of a 120 nm Au nanoparticle @ ~2 nm SiO_2 shell.

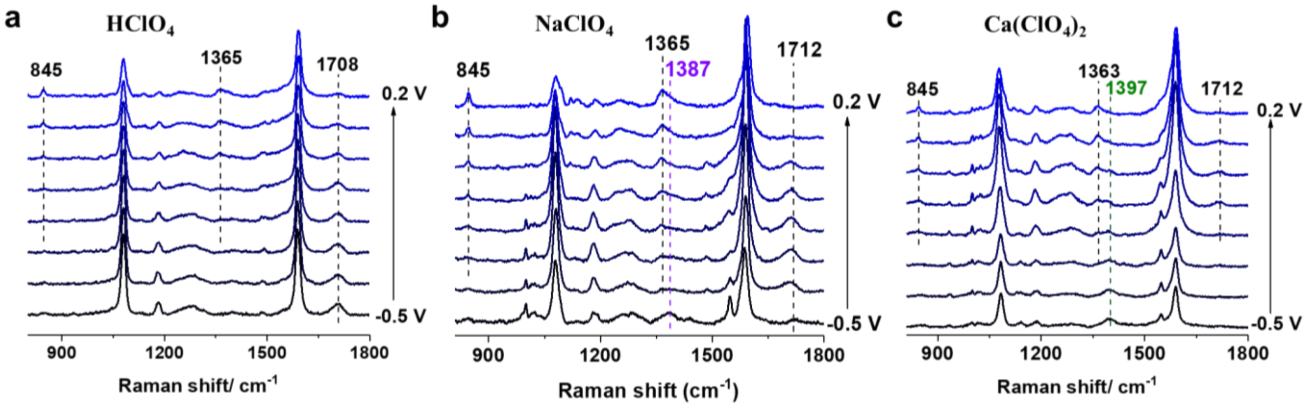

Supplementary Fig. 14 | In situ Raman measurements. Potential-dependent Raman spectra of 0.1 mM 4-MTBA obtained at Au(111) substrate in (a) 50 mM $HClO_4$, (b) 50 mM $NaClO_4$ and (c) 50 mM $Ca(ClO_4)_2$.

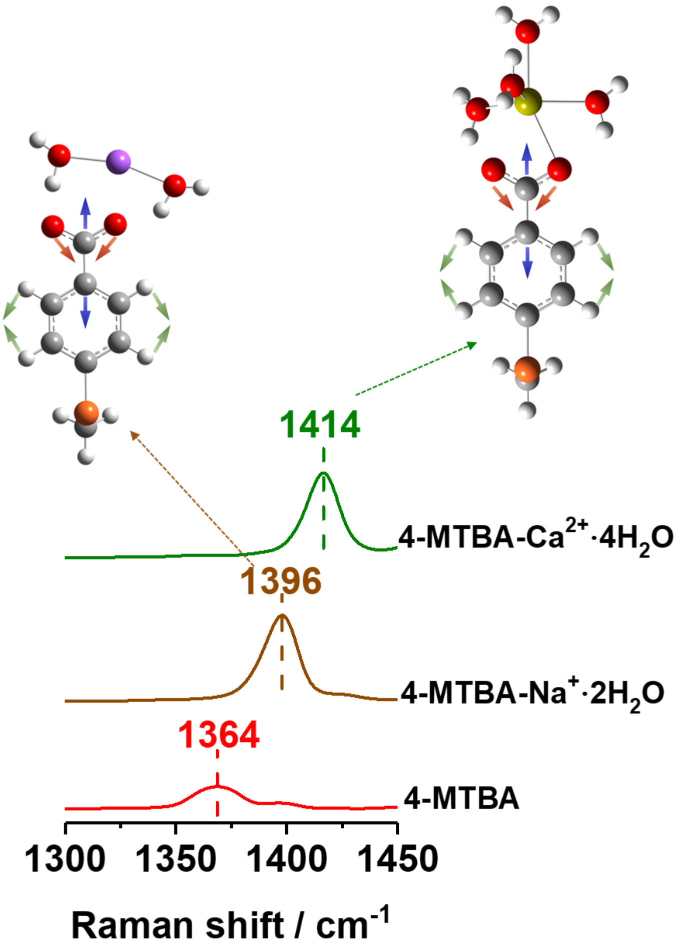

Supplementary Fig. 15 | Calculated Raman spectra. The calculated Raman spectra of investigated molecules. The fluorescent green, purple, orange, red, gray and white balls represent the Ca, Na, S, O, C and H atom respectively. Blue, green and red arrows point to the movement direction of corresponding O, C and H atoms at a transient moment.

All the calculated Raman spectra of investigated molecules were performed on the Gaussian 09 program[7]. As shown in Supplementary Fig. 15, the geometric structures were optimized using the B3LYP functional. For H, C, O, S, Na and Ca atoms, the 6-31+g** basis was adopted. Metal cations can be hydrated with different amounts of water molecules in an aqueous solution. According to previous reports, the Na^+ and Ca^{2+} can coordinate with three and six H_2O molecules[8,9]. Considering carboxyl–metal coordination complexation in binding configurations: unidentate for Na^+, bidentate for Ca^{2+} [10-13], we optimize structures of 4-MTBA with Na^+ ion and two water molecules, with the Ca^{2+} ion and four water molecules.

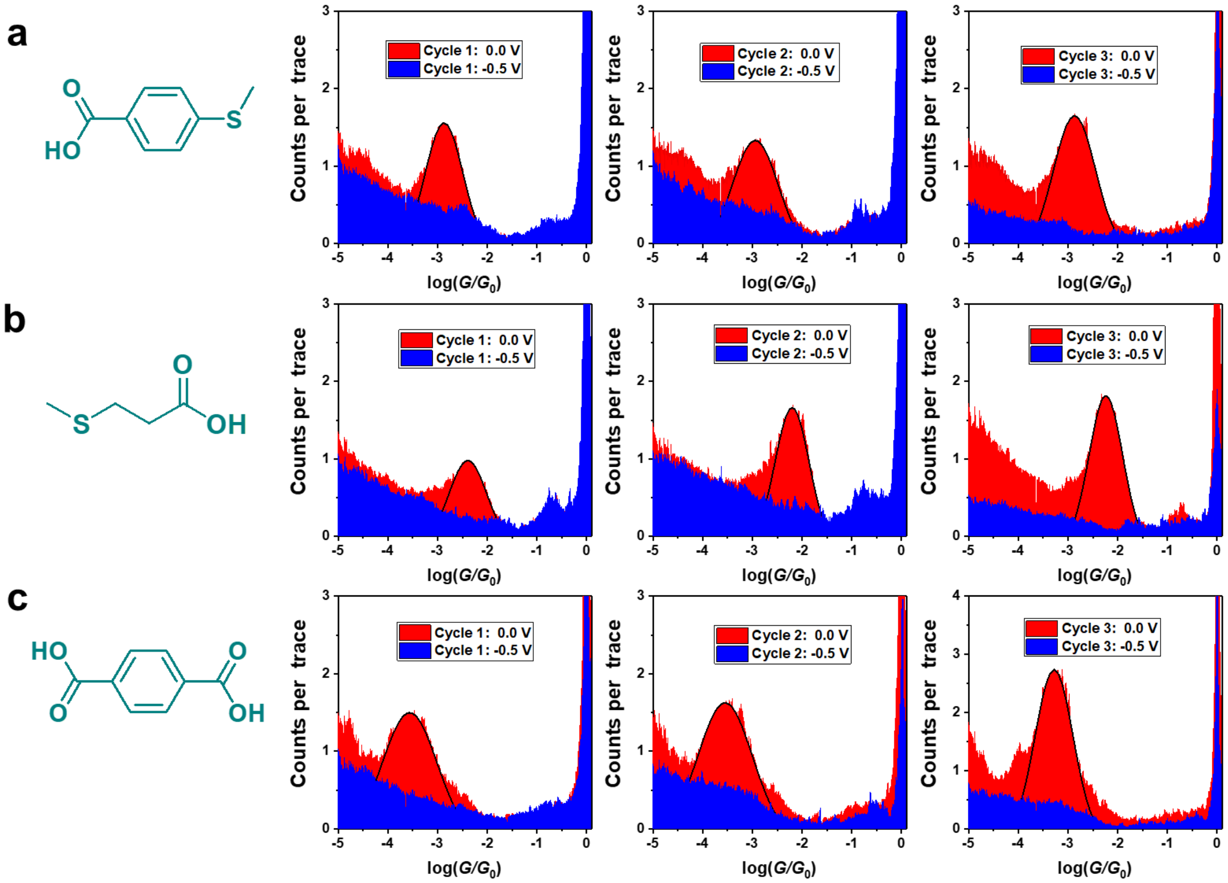

Supplementary Fig. 16 | Single-molecule switching performance. The 1D conductance histograms of (a) MPA and (b) TPA at -0.5 V and 0.0 V,respectively.

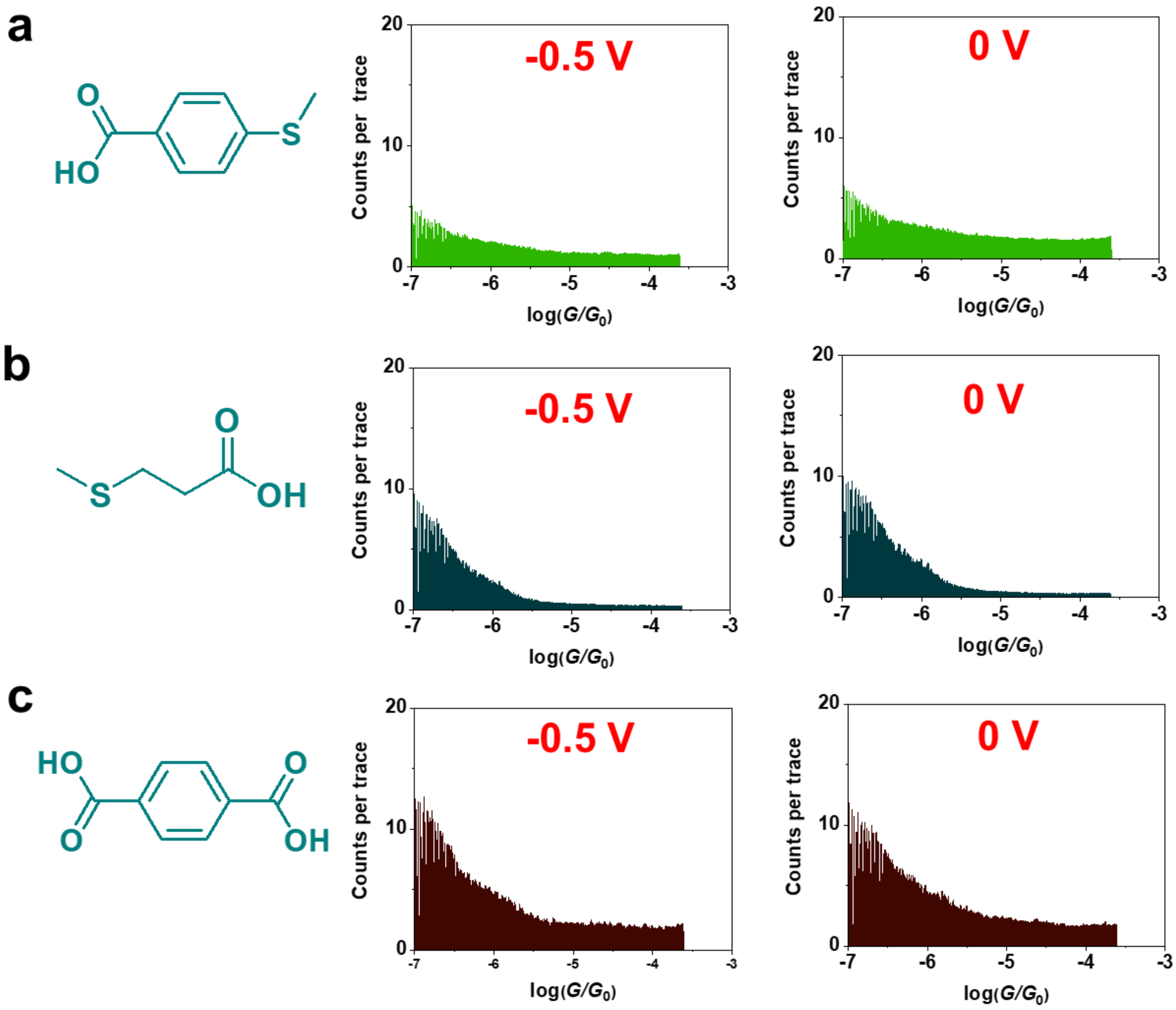

Supplementary Fig. 17 | Conductance measurements with lower single-range current amplifier. 1D conductance histograms of (a) MTBA, (b) TPA, and (c) MPA with lower single-range current amplifier at different potentials.

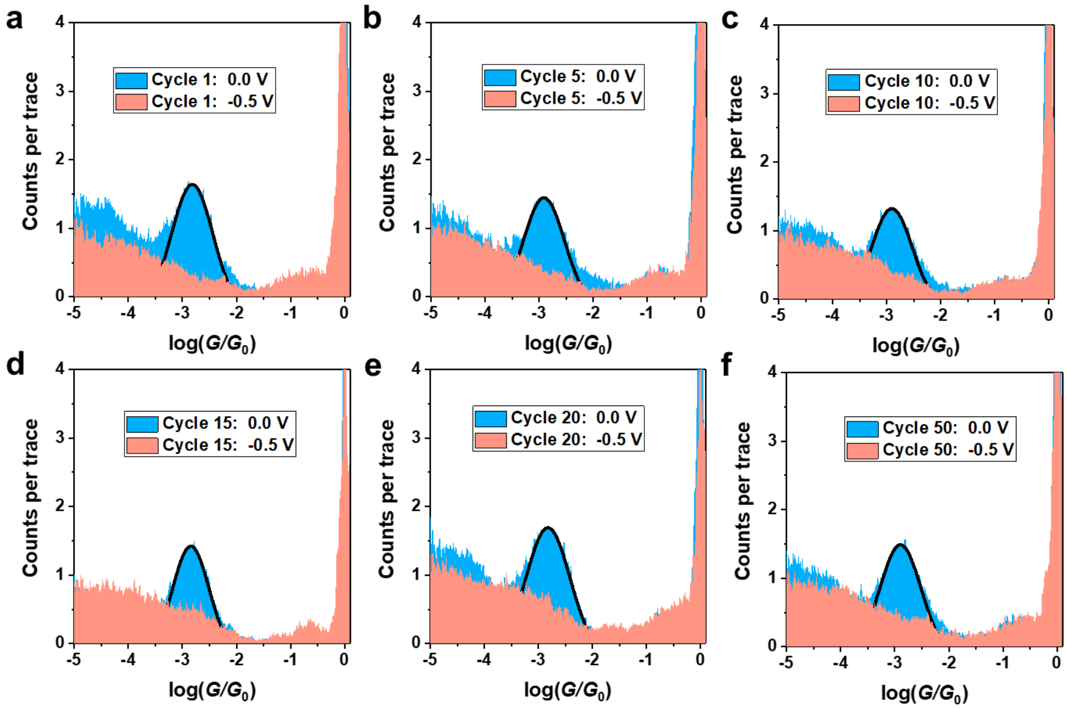

Supplementary Fig. 18 | Switch cycle test. The 1D conductance histograms after repeated cycle scanning of potentials between 0 and −0.5 V in cycles of (a)1, (b)5, (c)10, (d)15, (e)20, and (f)50.

We have performed the cyclic tests by repeatedly cyclically sweeping the potential between 0 and -0.5 V, followed by single-molecule conductance measurements. As shown in the Supplementary Fig. 18, the conductance peaks can repeatedly appear at 0 V and disappear at -0.5 V over 50 cycles in $NaClO_4$ solution. This demonstrates the good stability of localized cation-tuned reversible single-molecule switches in the electric double layer.

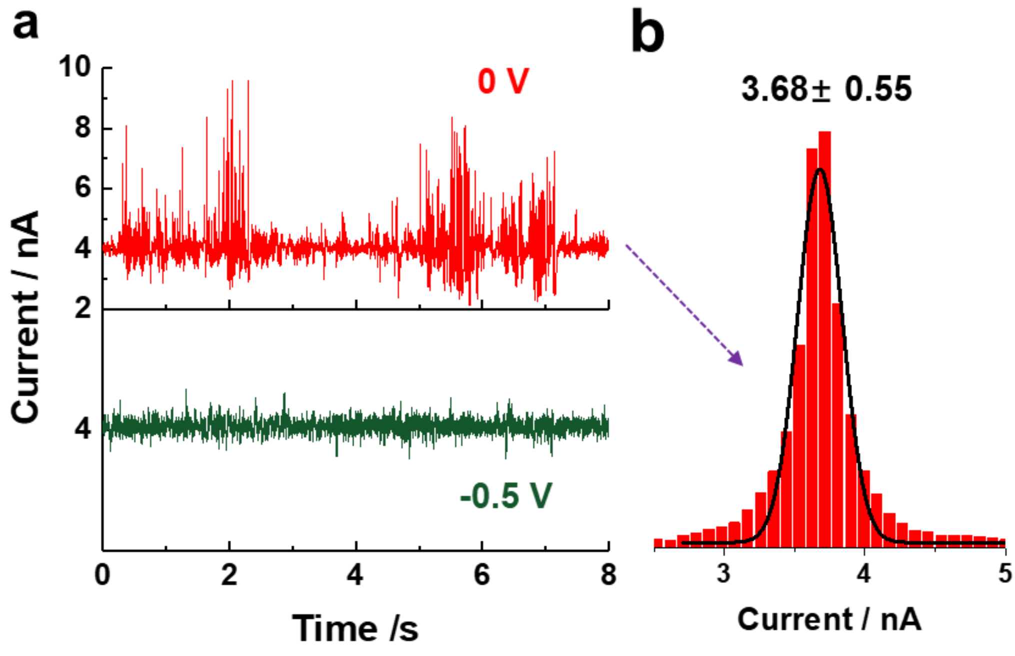

Supplementary Fig. 19 | I-t test with feedback loop. (a) Typical I-t curves in 0.1 mM 4-MTBA + 50 mM $NaClO_4$ solution with a very low STM feedback loop of 0.01 and current setpoint values of 4 nA at the substrate potentials of 0 and -0.5 V, respectively. (e) The corresponding distribution of current spikes heights in the I-t curve at 0.0 V.

The single-molecule conductance measurements using the I-t technique is briefly described as follows: Firstly, the Au tip is driven to approach the substrate until reaching the current value of 4 nA via piezoelectric control with a bias voltage of 50 mV at the substrate potential of 0 V, at which the molecular junction can be formed. The I-t curves are recorded with a very low STM feedback loop of 0.01 at the substrate potentials of 0 and -0.5 V.

The I(t) test with feedback loop at a fix bias can keep the relative distance between the tip and substrate by a preset current point. As the molecules are trapped into the nanogap between the two electrodes to form molecular junctions, the characteristic electron tunnel-current spikes can be observed on the I-t curves, which have been used to electronic single-molecule identification and sensors[14,15].Supplementary Fig. 19 proves the molecules can be trapped and tethered to two electrodes to form molecular junction at positively charged electrode surfaces, rather than at negatively charged electrode surface. Figure 5e is the statistics of relative height of current spikes in the I-t curves, all the current spikes with peak amplitude 1 nA above the baseline were characterized as signals.

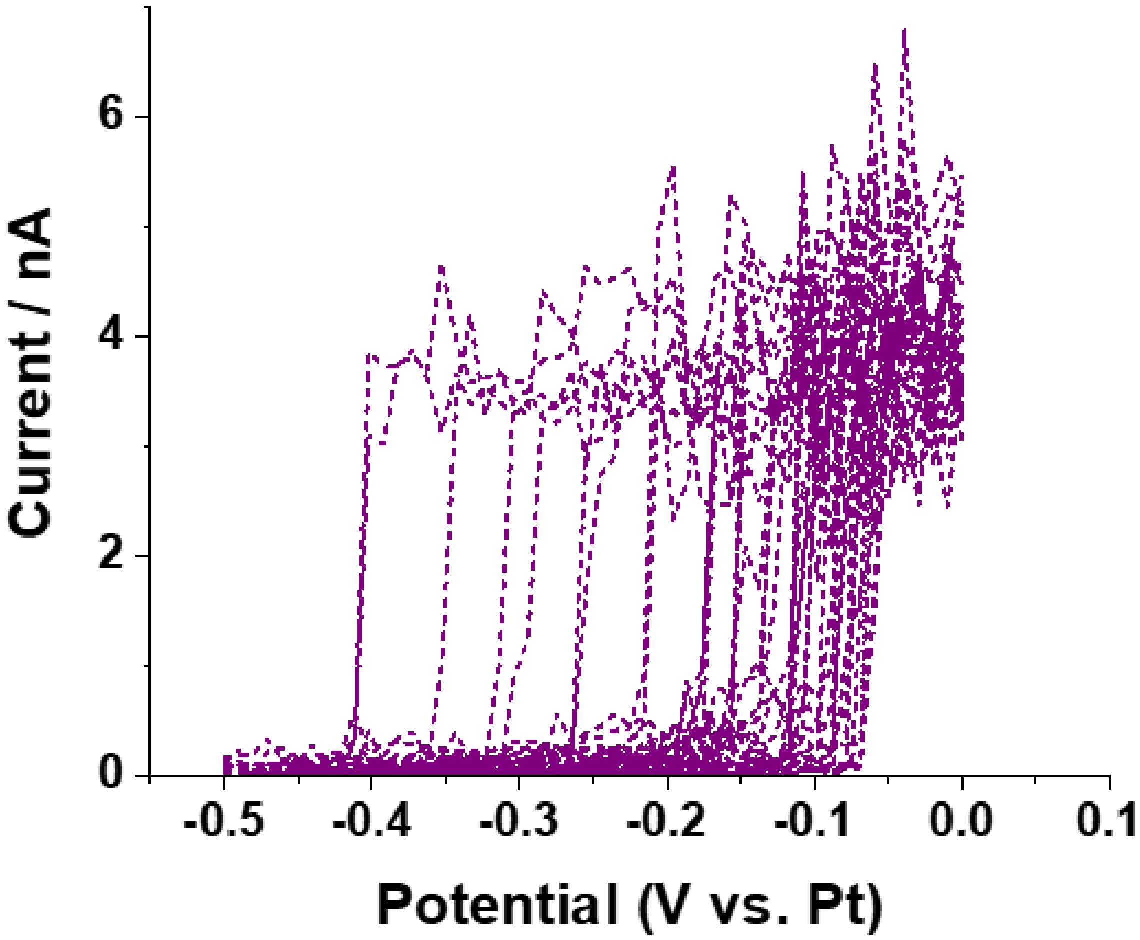

Supplementary Fig. 20 | I-V test. I-V curves obtained in 0.1 mM 4-MTBA + 50 mM $NaClO_4$ with a constant bias of 50 mV by simultaneously sweeping the tip and substrate potential from 0 and -0.5 V.

Supplementary Fig. 20 shows 42 traces of I-V curves recorded upon formation of molecular junctions. Obviously, when the gate potential is lower than −0.41 V, the tip current is turned off for all parallel tests, which is consistent with the disappearance of conductance peak below −0.4 V in single-molecule break junction experiments. These further prove the local-cation controlled single-molecule switch.

References

1 Li, J. F. *et al.* Surface analysis using shell-isolated nanoparticle-enhanced Raman spectroscopy. *Nat. Protoc.* **8**, 52-65 (2013).

2 Li, J. F. *et al.* Shell-isolated nanoparticle-enhanced Raman spectroscopy. *Nature* **464**, 392-395 (2010).

3 Wang, Y.-H. *et al.* In situ Raman spectroscopy reveals the structure and dissociation of interfacial water. *Nature* **600**, 81-85 (2021).

4 Borges, A., Xia, J., Liu, S. H., Venkataraman, L. & Solomon, G. C. The role of through-space interactions in modulating constructive and destructive interference effects in benzene. *Nano Lett.* **17**, 4436-4442 (2017).

5 Li, C. Z., Sha, H. & Tao, N. J. Adsorbate effect on conductance quantization in metallic nanowires. *Phys. Rev. B* **58**, 6775-6778 (1998).

6 Li, C., Mishchenko, A., Wandlowski, T. Charge transport in single molecular junctions at the solid/liquid interface. *Top. Curr. Chem.* **313**,121-188 (2011).

7 M. J. Frisch *et al.* Gaussian 09; Gaussian, Inc.: Wallingford, CT, USA, 2009.

8 Peng, J. *et al.* The effect of hydration number on the interfacial transport of sodium ions. *Nature* **557**, 701-705 (2018).

9 Zhao, W. *et al.* Evidence of formation of monolayer hydrated salts in nanopores. *J. Am. Chem. Soc.* **144**, 18976-18985 (2022).

10 Ho, W. K. H. *et al.* Probing conformation change and binding mode of metal ion–carboxyl coordination complex through resonant surface-enhanced Raman spectroscopy and density functional theory. *J. Phys. Chem. Lett.* **10**, 4692-4698 (2019).

11 Zhao, X. *et al.* Surface-enhanced Raman scattering optophysiology nanofibers for the detection of heavy metals in single breast cancer cells. *ACS Sens.* **6**, 1649-1662 (2021).

12 Tan, H. & Park, S.-Y. Poly(acrylic acid) hydrogel microspheres for a metal-ion sensor. *ACS Sens.* **6**, 1039-1048 (2021).

13 Ma, L. *et al.* Artificial monovalent metal ion-selective fluidic devices based on crown ether@metal–organic frameworks with subnanochannels. *ACS Appl. Mater. Interfaces.* **14**, 13611-13621 (2022).

14 Im, J. *et al.* Electronic single-molecule identification of carbohydrate isomers by recognition tunnelling. *Nat. Commun.* **7**, 13868 (2016).

15 Huang, S. *et al.* Identifying single bases in a DNA oligomer with electron tunnelling. *Nat. Nanotechnol.* **5**, 868-873 (2010).
